# Metacognitive preserved generation strategy benefits for both younger and elderly participants with schizophrenia

**DOI:** 10.1371/journal.pone.0241356

**Published:** 2020-11-20

**Authors:** Marie Izaute, Flavien Thuaire, Alain Méot, Fabien Rondepierre, Isabelle Jalenques

**Affiliations:** 1 Université Clermont Auvergne, CNRS, LAPSCO, Clermont-Ferrand, France; 2 Service de Psychiatrie de l’Adulte A et Psychologie Médicale, Centre Mémoire de Ressources et de Recherche, CHU Clermont-Ferrand, Clermont-Ferrand, France; 3 Service de Psychiatrie de l’Adulte A et Psychologie Médicale, Centre Mémoire de Ressources et de Recherche, CHU Clermont-Ferrand, Institut de Psychiatrie-GDR 3557, Université Clermont Auvergne, Clermont-Ferrand, France; Technion Israel Institute of Technology, ISRAEL

## Abstract

Cognitive memory and introspection disturbances are considered core features of schizophrenia. Moreover, it remains unclear whether or not participants with schizophrenia are more cognitively impaired with ageing than healthy participants. The aims of this study were to use a metacognitive approach to determine whether elderly participants with schizophrenia are able to improve their memory performance using a specific generation strategy and to evaluate the memory benefits for them using this strategy. 20 younger and 20 older participants with schizophrenia and their comparison participants matched for age, gender and education learned paired associates words with either reading or generation, rated judgment of learning (JOL) and performed cued recall. Participants with schizophrenia recalled fewer words than healthy comparison participants, but they benefited more from generation, and this difference was stable with ageing. Their JOL magnitude was lower than that of healthy comparison participants, but JOL accuracy was not affected by either age or the pathology. In spite of their memory deficit, elderly and younger participants with schizophrenia benefited remarkably from the memory generation strategy. This result gives some cause for optimism as to the possibility for participants with schizophrenia to reduce memory impairment if learning conditions lead them to encode deeply.

## 1. Introduction

Memory is acknowledged to be one of the cognitive functions most affected in schizophrenia [1–4] and cognitive disturbances are seen as better predictors of low functional outcome in participants with schizophrenia than clinical symptoms [5, 6]. Accordingly, improving their memory abilities should increase their functional outcome, and even small gains in their functioning or productivity could translate into large financial savings [7]. A number of neuroimaging studies support the view that high-level memory functions such as control over working memory [8], or semantic organization, proverb comprehension and inference of non-literal studies behind interlocutors’ use of proverbs are impaired in schizophrenia [9, 10]. In a review of neuroimaging studies conducted while participants with schizophrenia were performing executive tasks, Ranganath *et al*. [11] found that strategic memory encoding is linked to executive functions, and that both are impaired in schizophrenia. Even if executive functions impairment in participants with schizophrenia does not fully account for their memory impairment [12], it leads them to compensate with cognitive resources [13] and usually generates poor semantic performance [14].

Moreover, in addition to cognitive deficits [1, 11, 15–20] schizophrenia is often defined as a pathology of consciousness [21] and associated with a lack of self-awareness [22, 23]. Thus, as suggested by Bacon *et al*. [24], the concept of metamemory, which refers to monitoring of our awareness of our own memory capacity and control of related behaviour [25, 26], is of interest for gaining a better understanding of the cognitive behaviour of participants with schizophrenia. For example, during a learning memory task, monitoring refers to our own subjective assessment and is expressed as metamemory judgments (i.e., judgment of learning [27]). Control is the capacity to regulate our own cognitive behaviour, for example by allocating a given time to study [28] or by choosing an effective strategy. Thus, metamemory is central for learning strategy selection, cognitive resource allocation, and cognitive assessment of memory performance. The prerequisite for efficient monitoring is to be able to assess accurately whether or not a specific information answer is difficult or easy to learn, a process shown to be relatively intact in schizophrenia. Control of a learning task has been shown to be impaired [29, 30]. The strategic regulation of memory function in participants with schizophrenia is impaired not only at the time of retrieval of semantic knowledge, but also during encoding of episodic information. In a general knowledge task assessing semantic memory, Danion et al., (2001) [31] observed that participants with schizophrenia were able to take account of an incentive to obtain better accuracy. Indeed, they based their decisions to withhold or volunteer answers on previous monitoring and improved their memory performance. In a study on strategic control during information retrieval, Akdogan et al., (2014) [32] showed that participants with schizophrenia benefited from the strategic support. Participants with schizophrenia were less accurate than healthy participants when spontaneously reporting information from semantic memory. Nevertheless, when helped with contextual support, they were asked to respond either very precisely or by giving an interval response, their recall results were comparable to those of their healthy counterparts. Like healthy participants, participants with schizophrenia can strategically regulate their memory reporting when answers are to be provided within an experimentally fixed frame [32]. Another interesting finding from this study is that participants with schizophrenia present a deficit with self-initiation of strategy use [33, 34].

For episodic memory, when participants are instructed to learn pairs of words (cue-target), they are asked to predict the likelihood (Judgment of Learning) of recalling the target word during the memory test. These predictions (monitoring process) rely on a variety on contextual cues such as the difficulty of the word pair (association level), the number of times an item was presented, effortful processing during learning [35]. For participants from the general population, several studies have focused on a classic measure of metamemory control, namely self-paced study time. In the absence of time pressure, participants spend more time studying difficult items than easy ones [28]. For participants with schizophrenia, a significant dissociation was obtained between monitoring (relatively intact with sensitivity to contextual cues) and control (impaired during encoding episodic information) [29]. In this study, memory control was assessed using study-time allocation during learning, and memory monitoring was assessed using Judgments of Learning (JOL). During encoding of new information, participants with schizophrenia were able to adapt their JOL estimates to the frequency of item presentation. However, they did not adapt their study-time to this presentation frequency. There was no difference in the amount of study time allocated for an item presented once, twice, or three times. Nevertheless, according to the monitoring-affects-control hypothesis [36], another study [37] showed that when participants’ memory control follows the monitoring, they were able to adapt adequately their study time behaviour. When monitoring precedes the control of a learning phase schizophrenia participants are able to adapt their study time to the difficulty of pairs and spend more time re-studying the non-recalled answer during a second learning phase [37]. Thus metamemory monitoring remains accurate in schizophrenia, and metamemory control can be more efficient if some support is given to participants with schizophrenia.

A metacognitive intervention approach [38] is also essential for elderly people. Indeed, implementation of memory encoding and recovery strategies must be facilitated with age by means of tasks or instructions that will decrease the share of self-initiated processing. At one and the same time the elderly present both difficulties with implementing strategies suited to the nature of the task [39] and difficulties with adjusting their strategies [40]. Nevertheless, these effects of age may be reduced if older adults are encouraged or trained to use strategies. In a meta-analysis of the memory-improvement literature, Verhaeghen et al. (1992) [41] concluded that training older adults to use mnemonic strategies (e.g., imagery or the method of loci) can improve adults’ learning of new materials. For encoding, it is a matter of providing sufficient environmental support to direct learning towards the use of deep processing and encouraging the development of such processing on the information to be memorized (e.g., [38, 42]).

During encoding, other control metamemory processes can be evaluated like a specific memory strategy such as actively generating the to-be-remembered responses which usually produces a higher degree of learning than reading [43–47] and a higher accuracy of metamemory judgments [48]. This phenomenon (see for review, [49]), known as “the generation effect”, indicates the improvement in memory performance when participants generate the target word from a cue (lettuce—rab…) rather than just reading the word pair (lettuce—rabbit). This effect has been explained in terms of the active generation process itself which requires activating the semantic network between the cue presented at study and the target word to be generated. In their study, Froger et al. (2011) compared the perceived difficulty of the learning task by using reading and generation (learning strategy) in young and older adults [50]. Their results confirmed that all participants improved their memory performance in the generation condition. An additional aim of their study was to examine the effect of age on study-time allocation during learning strategies. Both groups perceived generation (estimate with overall prediction) as more difficult than reading as a learning strategy. Young participants allocated more time to the perceived difficulty strategy. Although older participants spent the same amount of time on average in both learning conditions (reading and generation), their memory performance was higher after the generation strategy. These results were interpreted as an impairment between young and older participants on control metacognitive process.

As functional capacity is related to neuropsychological abilities in older participants with schizophrenia [51, 52] a central question in schizophrenia is whether cognitive functioning deficits are due to ordinary ageing or degenerative ageing over the person’s life span [46, 53–57]. Since schizophrenia is seen as a variant of dementia [58], there has been considerable interest in ascertaining whether older adults with schizophrenia are particularly vulnerable to the cognitive effects of ageing [59]. Moreover, some studies suggest that cognitive performance is predictive of longevity in individuals with schizophrenia [60]. The growing number of studies involving ageing in participants with schizophrenia is a result of the lengthening of their life expectancy which, however, remains below that of the general population [61–64]. Understanding the interactions between aging and schizophrenia could provide an opportunity to better design age-sensitive intervention for remediation [52]. In a first pilot study to explore metamemory in elderly participants with schizophrenia [65] monitoring and control processes were evaluated. Participants had to learn pairs of words before assessing their JOLs taking into account the intrinsic characteristics of the material (strongly associated and non-associated word pairs) and then controlling their allocated re-study time in a second learning phase. Judgment of Learning (JOL) as an evaluation of monitoring was accurate, but memory control, as assessed by measuring study time allocation, was not as efficient for memory performance as in the case of healthy comparison participants.

To the best of our knowledge, there are no data available about how participants with schizophrenia and aging participants with schizophrenia cope with a mnesic strategy such as the generation effect. Several studies of the generation effect [42, 46, 47, 66, 67] found that generated words were recalled better than those that were read, even in older participants. Our aim was to examine the effectiveness of the generation strategy to evaluate the memory benefits for young and older adults with schizophrenia. We aimed to create a situation in which generation and reading strategy were implemented in a metacognition paradigm. The first goal of this study was to use a metacognitive approach to determine whether or not participants with schizophrenia are able to improve their memory performance using the generation strategy. Second, our goal was to estimate how participants were able to evaluate their JOL differently according to the difficulty of learning pairs of words and the two different strategies (generation versus reading). Learning difficulty was manipulated by the level of association between the two words of each pairs (weakly and strongly associated words). For metamemory, the level of association is typically regarded as an indicator of the level of difficulty of the material and therefore as an indicator of the evaluation of this difficulty. The Koriat model [68], for example, calls this characteristic an intrinsic cue, in respect of which other works have shown patients to be sensitive to this variation [29, 37]. We predict a replication of sensitivity to this difficulty in the case of participants with schizophrenia. They are able to estimate a higher JOL for the strongly associated pairs than for those whose association is weaker. Our third goal was to evaluate how participants with schizophrenia adapt the amount of time allocated to the study during learning.

## 2. Methods

### 2.1. Participants

This study was conducted in accordance with ethical standards and had the approval of the local ethics committee (Comité de Protection des Personnes Sud-Est 1, CHU Saint-Etienne, reference 2010–34). It was performed in accordance with relevant guidelines and was retrospectively registered in clinical trial the 9^th^ November 2017 (URL: https://clinicaltrials.gov/ct2/show/NCT03338179?term=NCT03338179&cntry1=EU%3AFR&rank=1, NCT03338179). Before the investigation started, all the participants and where appropriate their legal representatives provided written informed consent after receiving a full explanation of the study. As justification for the current sample size, to the best of our knowledge, not enough studies have been carried out to allow for an accurate assessment of the power analysis of the effect for participants with schizophrenia. Nevertheless, we did examine previous studies in the literature that used comparable stimuli [29, 31, 37, 69]. They show a deficit in metamemory judgment evaluation, and memory performance, in 18 to 23 participants with schizophrenia. As the task is very similar in terms of stimuli and the population studied, we think we need the same number of participants as previous experiments because the power of the effect ought to be very similar (similar population, similar procedure). For the comparison of elderly participants, only one previous study has been published with this type of paradigm for patients [65]. The group effect was obtained with groups of 13 participants. For the purposes of our study, 20 older (aged over 59 years and 6 months) and 20 younger (aged between 18 and 45 years) participants with schizophrenia living in the community, all of them clinically stable, were recruited from the Psychiatric Department of the University Hospital of Clermont-Ferrand. All of them met the criteria for schizophrenia as set out in the Diagnostic and Statistical Manual of Mental Disorders -IV-TR as determined by the consensus opinion of their current psychiatrist and a senior psychiatrist on the research team. The age of schizophrenia onset was under 40. Potential participants with any current co-morbid psychiatric disorder, including alcohol or substance abuse or dependence, were excluded from the study. Medicated participants with schizophrenia had been treated with stable doses of psychotropic medication for at least 4 weeks. 6 were taking typical neuroleptics, 23 atypical ones, and 10 a mixture of typical and atypical. One older participant with schizophrenia was neuroleptic-free. Their psychiatric symptoms were assessed according to the Positive and Negative Symptoms Scale (PANSS, [70]). Their IQ was assessed using a short version of the Wechsler Adult Intelligence Scale, revised (WAIS-R; [71]). Information processing speed was assessed with the digit/symbol subtest Wechsler Adult Intelligence Scale—WAIS-R [72] and letter comparison test (XO, [73]). Subjective memory complaint was assessed using participants’ responses to the Cognitive Difficulties Scale (see [74]).

Twenty older and 20 younger healthy participants matched with the participants with schizophrenia in terms of age, gender and level of education were also recruited. None of the 40 participants had a known neurological or psychiatric affection or suffered from current or past alcohol or substance abuse or dependence. Table 1 presents demographical, clinical and some descriptive statistics of group comparison data. There was no interaction effect between group and age.

**Table 1:** Demographic and clinical data for younger and older healthy comparison participants and participants with schizophrenia (standard deviations shown in brackets).

Participants	Older participants with schizophrenia n = 20	Younger participants with schizophrenia n = 20	Older healthy comparison participants n = 20	Younger healthy comparison participants n = 20
Men/women	12/8	12/8	12/8	12/8
Age (years)	63.7 (3.9)	31.6 (8.5)	64.0 (3.4)	32.4 (8.3)
Education level	11.5 (2.7)	12.0 (2.8)	11.5 (2.7)	12.6 (2.3)
Medication data				
Atypical neuroleptics	10	13		
Typical neuroleptics	4	2		
Atypical and typical neuroleptics	5	5		
None	1	0		
Onset of illness (years)	25.4 (4.8)	20.3 (4.4)		
PANSS total	67.5 (17.6)	69.9 (17.1)		
positive score	14.8 (6.2)	14.9 (3.4)		
negative score	19.2 (6.7)	20.4 (7.4)		
general psychopathology	33.5 (8.3)	34.6 (9.1)		
MMSE ^a^	27.1 (2.2)	27.3 (2.7)	28.8 (1.6)	29.3 (0.8)
HADS A ^a^	7.3 (3.5)	8.5 (3.6)	5.4 (3.0)	5.2 (2.5)
HADS D ^a^	5.3 (2.7)	5.5 (3.0)	3.4 (2.3)	2.2 (1.8)
IQ				
Verbal ^a^^b^	91.2 (13.4)	82.6 (18.9)	108.7 (13.0)	95.0 (15.6)
Performance ^a^^b^	101.0 (19.9)	90.9 (26.8)	130.9 (12.8)	105.7 (15.2)
Total ^a^^b^	95.1 (14.9)	86.1 (22.2)	120.8 (14.1)	99.8 (16.2)
MacNair ^a^	19.4 (6.6)	18.1 (7.3)	14.6 (6.2)	10.9 (3.5)
Digit/Symbol ^a^^b^	10.3 (3.9)	15.4 (4.2)	16.7 (3.3)	21.4 (3.9)
XO ^a^^b^	13.5 (6.8)	20.8 (6.6)	24.5 (4.0)	31.9 (5.1)

PANSS = Positive And Negative Symptom Scale; HADS = Hospital Anxiety and Depression Scale; IQ = Intelligence Quotient; Mac Nair: Subjective memory complaint was assessed using participants’ responses to the Cognitive Difficulties Scale; Digit/symbol subtest Wechsler Adult Intelligence Scale, XO = Letter comparison test.

^a^ significant group effect between participants with schizophrenia and comparison participants.

^b^ significant age effect.

### 2.2. Material

The items consisted of 28 weakly-associated word pairs (e.g. lettuce-rabbit) and 28 strongly-associated word pairs (e.g. watch-hour) from Ferrand and Alario [75]. Weakly-associated word pairs had an association value of less than 5 (M = 3.45; SD = 0.96), and strongly-associated word pairs a value higher than 25 (M = 42.81; SD = 16.03). Half of these weakly and strongly associated word pairs were randomly presented in the "reading" condition and the other half in the "generation" condition. The word pairs were then randomly divided into two lists (A and B). The two lists were counterbalanced across tests to avoid a list effect. There was no difference in association between the two lists for either weakly-associated, t(26) = .175, p = .86, or strongly-associated, t(26) = .721, p = .48, words. The experimental design was computerized, and the data were collected automatically.

### 2.3. Procedure

A computerized version of the tasks was used. The word pairs appeared on the screen one by one, and participants were instructed to read the pair or produce the target word (generation strategy) from the cue. They were then told that they could study each pair for as long as they liked during a maximum of 20 seconds. Participants controlled their own learning time, making it possible to measure their study-time allocation strategy For half of the word pairs participants had to read the pairs aloud, for the other half only the first three letters of the second word appeared on the screen, and participants had to generate the complete second word. After the learning phase, they had to perform a non-verbal distracting task during a 4-minute retention interval [76]. Then, the first item in each word pair (cue) was displayed on the screen without the second item (target). Using a 5-point scale (0%, 25%, 50%, 75% and 100%) participants were asked to give their JOL ratings, immediately following which there was a recall phase when each cue was presented, and they had to try to recall the target word. This procedure, which is easier for participants with schizophrenia, had been used already [65, 37]. Use of the same scale may allow the results obtained with this population to be compared whith those other strategies or memory indices.

### 2.4. Data analyses

All statistical analyses were run on SPSS Statistics. The significance level was set at p < .05. The analyses described in detail are those that were significant. All analyses of variance (ANOVAs) were computed with Group (healthy comparison participants or participants with schizophrenia) and Age (younger and older participants) as between-subject factors. The within-subject factors were Strategy (reading and generation) and item Association (weakly-associate and strongly associate). The dependent variables (see Table 2) were the percentage of words correctly generated, the percentage of read and generated words recalled for the memory performance analyses, the magnitude of JOL for the monitoring process, and the mean study time allocated for the control process. For the accuracy of metacognitive JOL monitoring in evaluating performance [77], the Goodmann-Kruskal gamma coefficient was calculated for each participant. This relative measure of correspondence, known as resolution or discrimination accuracy [78], refers to participants’ ability to discriminate between words that were recalled and those that were not. An outlier elimination rule was applied to all the analyses using the Tukey exclusion procedure. To avoid too much loss of power given the relatively low number of participants by groups, the Interquartile Range (IQR), the minimum distance below the first or above the third quartiles necessary to consider a data as outlier, was set at two. Only differences due to this elimination of outliers were reported.

**Table 2:** Mean percentage of generated items, correct recall, judgment of learning, mean study time (in seconds) and gamma coefficient for read and generated weakly associate and strongly associate items (standard deviations in brackets).

Participants	Older participants with schizophrenia n = 20	Younger participants with schizophrenia n = 20	Older healthy comparison participants n = 20	Younger healthy comparison participants n = 20
Generation					
Weakly-associate	81.8 (13.8)	84.7 (15.4)	89.0 (10.0)	92.2 (8.3)	
Strongly-associate	93.9 (10.2)	95.7 (7.1)	92.9 (16.7)	96.4 (5.9)	
Correct answer					
Generation					
Weakly-associate	47.5(21.9)	53.0 (22.2)	61.9 (18.8)	73.3 (17.0)	
Strongly-associate	71.4(17.3)	77.5 (16.6)	85.8 (11.3)	89.4 (10.1)	
Reading					
Weakly-associate	37.5 (16.2)	30.0 (20.7)	59.6 (18.3)	64.7 (19.5)	
Strongly-associate	58.6 (19.2)	63.60 (19.1)	81.4 (15.8)	88.2 (9.9)	
Judgment of learning					
Generation					
Weakly-associate	63.9 (22.4)	67.2 (17.2)	77.5 (15.1)	81.3 (12.1)	
Strongly-associate	72.3 (16.1)	76.2 (13.9)	89.3 (8.7)	90.8 (10.2)	
Reading					
Weakly-associate	54.2 (17.4)	49.9 (24.8)	71.5 (14.6)	73.9 (14.3)	
Strongly-associate	66.1 (21.9)	66.7 (19.1)	80.4 (16.3)	84.4 (11.3)	
Study time					
Generation					
Weakly-associate	11.6 (7.4)	9.6 (5.8)	6.3 (1.9)	7.9 (4.5)	
Strongly-associate	10.4 (7.8)	8.3 (5.9)	4.6 (1.7)	6.7 (4.7)	
Reading					
Weakly-associate	8.7 (6.2)	6.8 (5.4)	4.0 (1.3)	5.9 (4.6)	
Strongly-associate	8.6 (6.4)	6.2 (5.1)	3.8 (1.2)	5.3 (3.9)	
Gamma coefficient					
Generation	.85 (.14)	.81 (.20)	.84 (.18)	.91 (.12)	
Reading	.70 (.26)	.75 (.24)	.77 (.23)	.87 (.14)	

## 3. Results

Data used in this article are available at: https://osf.io/agzn7/.

### 3.1. Generation performance

A 2 (group: participants with schizophrenia, healthy comparison participants) x 2 (age: younger, older) x 2 (association: weakly-associate, strongly-associate) repeated-measures analysis of variance (ANOVA) was conducted on the percentage of words correctly generated. The results revealed a main effect of association, F(1,76) = 34.5, p < .001, η^2^_p_ = .312. Participants generate a higher percentage of answers with strong associates than with weak associates (respectively 94.7% and 86.9%). There was also an associate x group interaction F(1,76) = 7.9, p < .01, η^2^_p_ = .094. The difference in respect of generated items between participants with schizophrenia and healthy comparison participants appeared for weakly-associate items (respectively 83.2% and 90.6%) and not for strongly-associate items (respectively 94.8% and 94.7%). There were no effects of group, F(1,76) = 2.6, age, F(1,76) = 1.7, interaction group x age, F(1,76) = .06, age x association F(1,76) = .02, or group x age x association, F(1,76) = .07.

### 3.2. Memory performance

**Fig 1:**
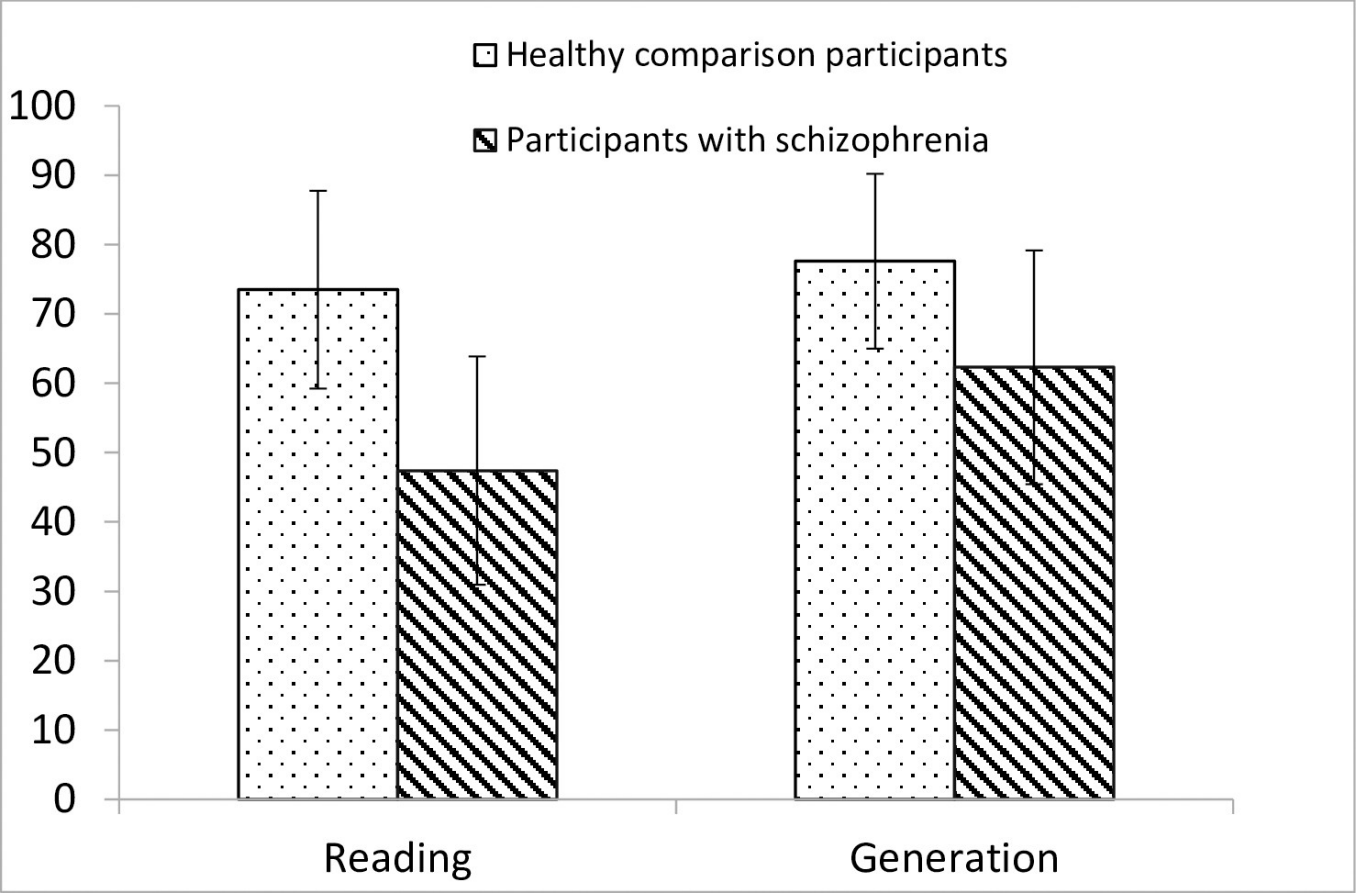
Mean percentages of correct answers obtained for participants with schizophrenia and healthy comparison participants as a function of strategy (reading and generation).

A 2 (group: participants with schizophrenia, healthy comparison participants) x 2 (age: younger, older) x 2 (strategy: reading, generation) x 2 (association: weakly-associate, strongly-associate) repeated-measures analysis of variance (ANOVA) was conducted on the percentage of read and generated words recalled (hereinafter referred to as “correct answers”). The results revealed a main effect of group, F(1,76) = 43.6, p < .001, η^2^_p_ = .364. Participants with schizophrenia gave a lower percentage of correct answers than healthy comparison participants (respectively 54.9% and 75.5%). There was also a main effect of strategy, F(1,76) = 50.3, p < .001, η^2^_p_ = .398. Participants gave a higher percentage of correct answers with the generation strategy than with the reading strategy (respectively 70.0% and 60.4%), suggesting that all participants benefited from the generation condition to enhance their memory performance. A main effect of association was obtained, F(1,76) = 243.9, p < .001, η^2^_p_ = .762. Participants gave a higher percentage of correct answers with strongly-associated words than with weakly-associated words (respectively 77.0% and 53.4%). Then, a strategy x group interaction F(1,76) = 16.3, p < .001, η^2^_p_ = .176 was obtained. Fig 1 shows the mean percentages of correct answers obtained for participants with schizophrenia and healthy comparison participants as a function of strategy. Scrutiny of this figure reveals that there was a memory advantage for generated items for both participants with schizophrenia and controls, but this advantage was bigger for participants with schizophrenia (47.4% for reading and 62.3% for generation) than for healthy controls (73.5% for reading and 77.6% for generation). This result suggests participants with schizophrenia benefit more from generation than healthy controls. This is true for both young and older adults. There were no effects of age, F(1,76) = 2.1, interaction group x age, F(1,76) = .5, age x strategy F(1,76) = 2.5, group x age x strategy F(1,76) = 1.0, group x associate F(1,76) = 2.2, age x associate F(1,76) = .4, associate x strategy F(1,76) = 1.5, group x association x strategy, F(1,76) = .01, or group x age x association x strategy, F(1,76) = .06.

To assess whether the difference obtained in terms of the percentage of correctly generated words altered the mnesic performance effects, an ANCOVA was performed. This analysis yielded the same results, namely that the percentage of words recalled did not depend on the percentage of words generated. Thus, the strategy x group interaction and next strategy x association x age interaction cannot be attributed to the percentage of generated words.

**Fig 2:**
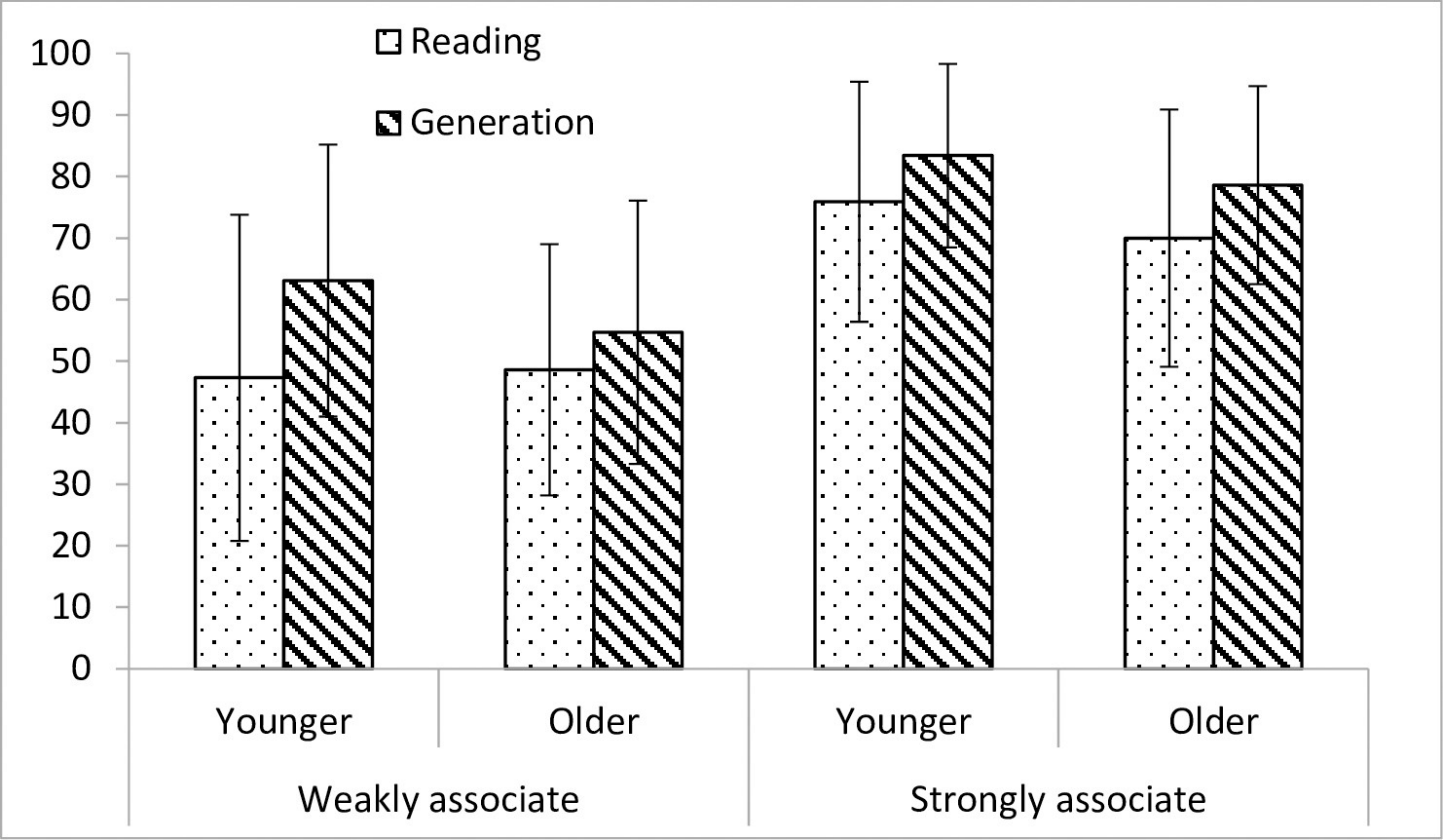
Mean percentages of correct answers obtained for younger and older participants as a function of strategy (reading and generation) and association (weakly associate and strongly associate).

Finally, a strategy x association x age interaction, F(1,76) = 5.0, p < .05, η^2^_p_ = .061, was also obtained. Fig 2 shows the mean percentages of correct answers obtained for younger and older participants as a function of strategy and association.

Scrutiny of this figure reveals that there was no difference between younger and older participants as regards the increase from reading to generation with strongly associated items (respectively +7.5% for younger participants and +8.6% for older participants). For weakly associated items, however, the difference between reading and generation was greater for younger participants (+15.8%) than for older participants (+6.1%).

### 3.3. Metamemory monitoring

The same ANOVA was conducted on JOL ratings for all the answers. The results revealed a main effect of group, F(1,76) = 29.7, p < .001, η^2^_p_ = .281. Participants with schizophrenia estimated lower JOL ratings than healthy comparison participants (respectively 64.6% and 81.1%). There was also a main effect of strategy, F(1,76) = 41.8, p < .001, η^2^_p_ = .355. Participants estimated higher JOL ratings with generation strategy than with reading strategy (respectively 77.3% and 67.4%). Finally, there was a main effect of association, F(1,76) = 89.0, p < .001, η^2^_p_ = .539. Participants estimated higher JOL ratings with strongly-associated items than with weakly-associated items (respectively 78.3% and 67.4%). There were no effects of age, F(1,76) = .4, interaction group x age, F(1,76) = .1, group x strategy F(1,76) = 1.6, age x strategy F(1,76) = .8, group x age x strategy F(1,76) = .6, group x associate F(1,76) = 2.2, age x associate F(1,76) = .3, group x age x associate F(1,76) = .5, associate x strategy F(1,76) = 1.1, group x association x strategy, F(1,76) = 2.2, age x association x strategy, F(1,76) = .9, or group x age x association x strategy, F(1,76) = .002.

### 3.4. Relative correspondence between memory performance and judgment of learning: Gamma coefficient

In metamemory two measures were usually used: calibration (measure of absolute accuracy) and gamma (measure of relative accuracy, [79]). The value of the relative measurement shows that the relative precision (gamma) of participants with schizophrenia is very high, signalling that they are able to discriminate between items they recall and those they do not recall. The gamma coefficient (see Table 2) has to be calculated individually for each participant. The values of the gamma coefficient can range from 1.0 (full agreement between confidence level and answer provided) to -1.0 (complete disagreement between the confidence level rating and answer provided).

A 2 (group: participants with schizophrenia, healthy comparison participants) x 2 (age: younger, older) x 2 (strategy: reading, generation) ANOVA was performed on this gamma coefficient. Excluding outliers resulted in a significant main effect of the strategy F(1,68) = 10.61, p < .01, η^2^_p_ = .135, with gamma values higher for the generation (.86) task than the reading task (.78), and no interaction effect, indicating that there was no difference in accuracy between younger and older participants or between participants with schizophrenia and healthy comparison participants in this respect. High gamma coefficients indicated that metamemory judgments closely matched true memory performance both for participants with schizophrenia and healthy comparison participants and both for both younger and older participants. There were no effects of group, F(1,76) = .3, age, F(1,75) = 2.9, interaction group x age, F(1,75) = .2, strategy F(1,73) = .3, group x strategy F(1,73) = 1.0, age x strategy F(1,76) = .3, or group x age x strategy F(1,76) = .0002.

### 3.5. Allocation of study time

A 2 (group: participants with schizophrenia, healthy comparison participants) x 2 (age: younger, older) x 2 (strategy: reading, generation) x 2 (association: weakly-associate, strongly-associate) ANOVA was conducted on time allocation in respect of all the answers. The results revealed a main effect of group, F(1,76) = 8.4, p < .01, η^2^_p_ = .100. Participants with schizophrenia allocated more time to study than healthy comparison participants (respectively 8.8s and 5.5s). There was a main effect of strategy, F(1,76) = 173.2, p < .001, η^2^_p_ = .695. Participants allocated a longer study time to the generation strategy than the reading strategy (respectively 8.2s and 6.2s). There was also a main effect of association, F(1,76) = 83.8, p < .001, η^2^_p_ = .524. Participants allocated a longer study time to weakly associated items than strongly-associated items (respectively 7.6s and 6.7s). A strategy x group interaction, F(1,76) = 6.8, p < .05, η^2^_p_ = .083, was significant, suggesting that the difference between the two strategies (more time for generation than for reading) was greater for participants with schizophrenia (respectively 10.0s and 7.6s) than for healthy comparison participants (respectively 6.4s and 4.7s). Finally, a strategy x association interaction F(1,76) = 24.9, p < .001, η^2^_p_ = .247 was obtained. More time was allocated for weakly associate items than for strongly-associate items in generation strategy (respectively for weakly-associate items 8.8s and for strongly-associate 7.5s). There was no difference between these two types of items for reading (respectively, 6.4s and 6s). The same results were obtained with outliers excluded (see the elimination rule in the data analysis) except that the group x age interaction became significant, F(1, 71) = 5.28, p < .05, η^2^_p_ = .069, indicating a greater difference between participants with schizophrenia and healthy participants for the elderly (respectively, 9.8s and 4.6s for the older and 6.2s and 5.1s for the younger participants). There were no effects of age, F(1,76) = .2, interaction group x age, F(1,76) = 3.1, age x strategy F(1,76) = .1, group x age x strategy F(1,76) = .02, group x associate F(1,76) = .4, age x associate F(1,76) = .6, p>.1, group x age x associate F(1,76) = 1.2, group x association x strategy, F(1,76) = .3, age x association x strategy, F(1,76) = 3.7, or group x age x association x strategy, F(1,76) = 1.1.

## 4. Discussion

The aims of this study were to examine a specific generation strategy to assess its memory benefits for young and older adults with schizophrenia, and to use a metacognitive approach to determine whether or not participants with schizophrenia are able to improve their memory performance with the help of this generation strategy. The results show that memory performance was lower for both groups with schizophrenia than for both healthy groups. This finding is consistent with evidence which has repeatedly indicated that episodic memory is defective in schizophrenia [1, 37, 80–83]. Nevertheless, our results replicated earlier findings that generation improves the memory performance of both young and older adults [50, 84]. To the best of our knowledge, this is the first time a study has shown that participants with schizophrenia benefited more from the generation strategy than healthy comparison participants. Even if memory performance of participants with schizophrenia is still lower than that of healthy comparison participants, the improvement in memory performance with the generation strategy is greater in the case of participants with schizophrenia than healthy comparison participants. This is in contradiction with Iddon et al. (1998) [85], according to whom participants with schizophrenia failed to benefit from strategy selection. In their paradigm, participants had to discover the category exemplar strategy and then apply it. This procedure required self-initiation of the strategy, which is known to be impaired in schizophrenia [33, 34]. Our results indicate that providing cognitive support with generation at encoding helped participants with schizophrenia reduce the gap with comparison participants with respect to memory performance; this result is consistent with the findings of Thuaire et al. 2012 [37]. When the paradigm allows for it and accompanies participants with schizophrenia in the implementation of adapted control, they adapt their study time more precisely and improve memory performance. Indeed, based on their experience, participants benefited from a first attempt at recall, showing that they were able to adapt their study time to the difficulty of pairs and spend more time re-studying the non-recalled answer during a second learning phase. In the same vein, regarding the retrieval process, Akdogan et al. (2014) [32] showed that participants with schizophrenia benefited remarkably from the framing of responding. The same result was obtained with older participants, whose memory performance improved when ‘appropriate environmental support’ was provided [86]. Our study is original in that it examined the generation strategy as a possible remediation tool for participants with schizophrenia as well as elderly participants. The results we obtained with use of a generation strategy indicate that younger and older participants with schizophrenia reap the same benefits as their healthy counterparts. This is consistent with Taconnat and Isingrini (2004) [66], who showed that young, older, and very old participants benefited equally from this strategy. Thus, the present experiment extends the latter result to young and older participants with schizophrenia.

A closer look at our results reveals that mnesic performance is better for stronger associates than weaker associates. However, the improvement with weak associates is greater for generation strategy than reading strategy only in the case of younger participants (healthy and with schizophrenia alike). The same effect was obtained by Taconnat et al. 2008 [42], but only with healthy participants. Our results extend this effect to schizophrenia participants. When the generation strategy is structured with the first letters of the search word, participants use and benefit from using this strategy. Guerrero et al. (2019) [86] suggest it is the implementation of effective encoding processes that accounts for the generation effect. Such efficiency would depend on metamemory accuracy and the capacity to self-initiate internal strategies. Older adults were also less spontaneously aware that generation led to better memory performance [86]. We evaluated this awareness by monitoring judgment of learning. With our results, according to the difficulty of the material to be learned, we observed the classic adaptation in metamemory monitoring results observed with other metamemory paradigms [28]. As with younger participants with schizophrenia, the JOL ratings of elderly participants with schizophrenia were lower overall than those of the healthy participants, corresponding to their lower memory performance. Nevertheless, both groups of participants with schizophrenia were equally as able as their healthy counterparts to assign lower JOLs adequately to weakly-associate word pairs as opposed to strongly-associate word pairs. This finding confirms previous observations [29, 31, 37] which had shown, first, that monitoring by participants with schizophrenia accurately reflects their memory performance. Secondly, participants with schizophrenia were equally as able as their healthy counterparts to attribute JOLs as a function of item difficulty. Third, both schizophrenia groups estimated higher and more accurate JOLs for generation strategy [48] than for reading. Previous studies have shown that their monitoring is also sensitive to cues like item repetition when they make their JOL [29], as well as to partial information about the memory target [69]. Monitoring by participants with schizophrenia therefore seems to be sensitive to the cues provided by the conditions of the memory task, with the basis for monitoring [68] appearing to be relatively intact in participants with schizophrenia. Finally, both schizophrenia groups gave more accurate JOLs in the generation condition, thus extending previous results with healthy participants [48] to participants with schizophrenia. Most studies about metamemory monitoring in ageing have focused on feeling of knowing (FOK) judgments and have found that such judgments are impaired in older participants [87]. However, this impairment seems to be linked to a difficulty among with elderly with retrieving contextual information [88]. Thus, JOLs, which are not based on such contextual information, are not impaired with ageing [89], and older participants show a delayed-JOL effect equivalent to that observed with younger participants [90]. In accordance with these studies and with the preservation of monitoring in schizophrenia [29, 69], older participants with schizophrenia were able to adapt their JOLs to the difficulty of the materials and to the encoding strategy in order to produce accurate judgments [65]. Procedures based on metamemory monitoring and control have been shown to be efficient in older adults [38], who have been observed to improve the effectiveness of learning by accurately monitoring their progress towards a learning goal and by using the monitoring output to allocate study time appropriately. In our procedure, study time is allocated before JOL monitoring. Both participants with schizophrenia and healthy controls were able to adapt their study time during generation strategy: more time was allocated for weakly-associated items than for strongly associated items. However, there was no difference between these two types of items for reading. For all participants, more time was allocated for generated items than for reading. Our results corroborate those of Matvey et al. (2001) [91] who found that participants considered memory was enhanced more by generating than by reading words pairs. In our results, the mnesic improvement with generation is greater for participants with schizophrenia than for healthy comparison participants. Nevertheless, their JOL were not sensitive to this bigger improvement, which reflects the corresponding mnesic results we obtained. Participants with schizophrenia benefit more from the generation strategy than their healthy counterparts. In terms of the strategic control of learning, our results contradict those of Bacon et al., (2007) [29], who found that participants with schizophrenia memory control was impaired because they did not adapt their study time to the frequency of item presentation. In our results, as in typical results [28] and those obtained with participants with schizophrenia [37], all participants allocated more study time to difficult items than easy items. As regards generation, our results contradict those of Froger et al. (2011) who found that older adults took the same time to study the generated and read target words whereas younger adults spent longer on the generation task [50]. In our results, all participants allocated more time to the generation strategy compared to the reading strategy. However, like in our study, despite the differences in study time, there was a similar generation effect on memory performance.

A number of limitations need to be considered when interpreting these results. A first limitation of our study is that most of the participants with schizophrenia were integrated in the community which is known to be an important component of welfare. Another limitation is the nature of the task that had the advantage of examining the amount of generation effect on word pairs in a experimentally controlled study but it was not very ecological memory. More research, with more comprehensive testing, therefore seems necessary. A third limitation is the relatively small samples. Nevertheless, the effect size of the group difference in the generation condition was robust. Moreover, it is important to stress that each patient was matched with one healthy comparison participant in terms of age, gender and level of education. All of our participants with schizophrenia were chronic, medicated treated according to the guidelines for biological treatment of schizophrenia of the World Federation of Societies of Biological Psychiatry (WFSBP, [92]). Therefore, the potential effects of antipsychotic medication cannot be ruled out. It is very unlikely, however, that antipsychotic drugs were responsible for the enhanced memory manipulation observed in schizophrenia [93, 94]. Second, we found there to be no difference between older and younger participants with schizophrenia in respect of memory and metacognitive monitoring and control aspects. This result was confirmed by neuropsychological measurements in which only the two subtests served to assess processing speed (Digit/symbol subtest, Letter comparison test), with older participants performing worse than younger participants. The way the participants were treated, in line with the guidelines, meant it was possible to limit negative side-effects and cognitive decline in the case of elderly participants with schizophrenia.

In conclusion, this study puts forward some original findings. In spite of their memory deficit, older and younger participants with schizophrenia benefited remarkably from the memory generation strategy. Use of efficient memory strategies could contribute to memory rehabilitation. This result gives some cause for optimism as to the possibility for participants with schizophrenia to reduce their memory impairment if learning conditions cause them to encode deeply.
